# Novel *PANK2* Mutations in Patients With Pantothenate Kinase-Associated Neurodegeneration and the Genotype–Phenotype Correlation

**DOI:** 10.3389/fnagi.2022.848919

**Published:** 2022-04-06

**Authors:** Wen-Bin Li, Nan-Xiang Shen, Chao Zhang, Huan-Cheng Xie, Zong-Yan Li, Li Cao, Li-Zhi Chen, Yuan-jin Zeng, Cui-Xia Fan, Qian Chen, Yi-Wu Shi, Xing-Wang Song

**Affiliations:** ^1^Department of Neurology, Institute of Neuroscience, Second Affiliated Hospital of Guangzhou Medical University, Guangzhou, China; ^2^Key Laboratory of Neurogenetics and Channelopathies of Guangdong Province and the Ministry of Education of China, Guangzhou, China; ^3^Suzhou Hospital of Anhui Medical University (Suzhou Municipal Hospital of Anhui Province), Suzhou, China; ^4^Department of Neurology, Shanghai Jiao Tong University Affiliated Sixth People’s Hospital, Shanghai, China

**Keywords:** *PANK2*, mutation, mitochondria, severity, pKAN

## Abstract

Pantothenate kinase-associated neurodegeneration (PKAN) is a rare genetic disorder caused by mutations in the mitochondrial pantothenate kinase 2 (*PANK2*) gene and displays an inherited autosomal recessive pattern. In this study, we identified eight *PANK2* mutations, including three novel mutations (c.1103A > G/p.D368G, c.1696C > G/p.L566V, and c.1470delC/p.R490fs494X), in seven unrelated families with PKAN. All the patients showed an eye-of-the-tiger sign on the MRI, six of seven patients had dystonia, and two of seven patients had Parkinsonism. Biallelic mutations of *PANK2* decreased PANK2 protein expression and reduced mitochondrial membrane potential in human embryonic kidney (HEK) 293T cells. The biallelic mutations from patients with early-onset PKAN, a severity phenotype, showed decreased mitochondrial membrane potential more than that from late-onset patients. We systematically reviewed all the reported patients with PKAN with *PANK2* mutations. The results indicated that the early-onset patients carried a significantly higher frequency of biallelic loss-of-function (LoF) mutations compared to late-onset patients. In general, patients with LoF mutations showed more severe phenotypes, including earlier onset age and loss of gait. Although there was no significant difference in the frequency of biallelic missense mutations between the early-onset and late-onset patients, we found that patients with missense mutations in the mitochondrial trafficking domain (transit peptide/mitochondrial domain) of PANK2 exhibited the earliest onset age when compared to patients with mutations in the other two domains. Taken together, this study reports three novel mutations and indicates a correlation between the phenotype and mitochondrial dysfunction. This provides new insight for evaluating the clinical severity of patients based on the degree of mitochondrial dysfunction and suggests genetic counseling not just generalized identification of mutated *PANK2* in clinics.

## Introduction

Pantothenate kinase-associated neurodegeneration (PKAN) (OMIM #234200), a subtype of neurodegeneration defined as brain iron accumulation (NBIA) disorders, is characterized by the accumulation of iron in the basal ganglia (Gregory et al., 2009). PKAN frequently manifests as severe dystonia, young-onset Parkinsonism, pigmented retinopathy, and loss of movement control (Hayflick et al., 2003). Based on the onset age, it is classified into the two groups: early onset (<10 years old when first symptoms start), otherwise known as classic onset, and late onset (≥10 years old when symptoms start), otherwise known as atypical onset. Patients with early-onset PKAN show rapid disease progression, loss of ambulation approximately 15 years after the first symptoms, and tend to develop pigmentary retinopathy. Those with later onset show slower progression, maintain independent ambulation for more than 15 years after first symptoms, and tend to have speech disorders and psychiatric features (Pellecchia et al., 2005). Most patients have the eye-of-the-tiger sign on brain MRI (McNeill et al., 2008) and display inherited mutations of pantothenate kinase 2 (*PANK2*) gene (OMIM *606157) in an autosomal recessive pattern (Zhou et al., 2001).

Pantothenate kinase 2 is located on chromosome 20p13 and encodes the PANK2 protein consisting of 570 amino acids. PANK2 belongs to the pantothenate kinase family (PANK1–4) and is the only pantothenate kinase located in the mitochondria (Prokisch and Meitinger, 2003). It catalyzes the biosynthesis of coenzyme A (CoA) and acts as a rate-controlling enzyme in the first step of the CoA biosynthesis pathway (Begley et al., 2001). CoA is a key molecule for hundreds of metabolic reactions, including the tricarboxylic acid cycle and neurotransmitter synthesis (Leonardi et al., 2005), and dysfunction is associated with neurodegeneration with brain iron accumulation (Srinivasan et al., 2015). PANK2 is comprised of a transit peptide/mitochondrial (TPM) (1–45 aa) domain at the N-terminal region, intermediate/regulatory (I/R) domain (47–211 aa) in the central region, and PANK catalytic core domain (CCR) (212–570 aa) at the C-terminal region (Zhang et al., 2006). The location of PANK2 in mitochondria is vital for regulating CoA biosynthesis (Leonardi et al., 2007). To date, more than 100 mutations have been reported with different mutation types and locations in *PANK2* (Chang et al., 2020). Mutations in *PANK2* have been reported to disrupt mitochondrial function, including increased oxidative status, disturbed CoA metabolism, and iron homeostasis, which is associated with PKAN (Brunetti et al., 2012; Campanella et al., 2012; Jeong et al., 2019). Mitochondria impairment is related to many neurodegenerative disorders, such as Parkinson’s disease and Alzheimer’s disease (Burté et al., 2015; Que et al., 2021; Wang et al., 2021a). However, the mitochondrial functional alteration caused by biallelic *PANK2* mutations and the potential relationship with the severity of phenotype is still unknown. In this study, we reported seven biallelic PANK2 mutations from seven unrelated families, including three novel mutations, c.1103A > G/p.D368G, c.1696C > G/p.L566V, and c.1470delC/p.R490fs494X. All the biallelic mutations derived from patients reduced mitochondrial membrane potential (MMP) in human embryonic kidney (HEK) 293T cells, which correlated to the severity of the phenotype in the patients. We also systematically reviewed all the reported patients with biallelic *PANK2* mutations and found that patients with loss-of-function (LoF) mutations or missenses in their TPM domain had more severe phenotypes. These results showed a potential relationship between the phenotype and mitochondrial dysfunction, suggesting that genetic counseling should consider the degree of mitochondrial dysfunction caused by the biallelic mutation.

## Materials and Methods

### Inclusion of Patients

All the patients were recruited from the genetic outpatient department of the Second Affiliated Hospital of Guangzhou Medical University, including three patients transferred from the Suzhou Hospital of Anhui Medical University, Department of Neurology, and Shanghai Jiao Tong University Affiliated Sixth People’s Hospital. Brain MRI scans and detailed clinical data were collected, including age at onset, gait disturbance (GD), general and neurological examination results (dystonia, tremor, chorea, dysarthria, dysphagia, cognitive decline, and pyramidal signs), sex, and age. Genomic DNA of peripheral blood was extracted from the patients and their parents (Qiagen, Hilden, Germany) for sequencing.

Mutation screening of *PANK2* was performed using Sanger sequencing. Patients were classified as early-onset or late-onset atypical types (Pellecchia et al., 2005). This study was approved by the Medical Ethics Committee of Second Affiliated Hospital of Guangzhou Medical University. Written informed consent was obtained from the patients and their parents (for children).

### Recombinant Plasmid Construction

Human *PANK2* complementary DNA (cDNA) (NM_153638.4) was amplified by PCR (forward primers: 5′-TCTCGAGCTCAAGCGCTAGCTGCCACCATGAGGAGG CTC-3′, reverse primer: 5′-ATAAGCTTGATATCGAATTC TCACGGGATCTTCAACAGCT-3′) and cloned into the flap-Ub promoter-GFP-WRE (FUGW)-enhanced green fluorescent protein (EGFP) plasmid using the ClonExpress Kit (Vazyme, cll3, China). The FUGW-PANK2-EGFP product was confirmed by Sanger sequencing. *PANK2* missense variants, D324Y, D368G, D378G, D452G, N500I, and L566V, were generated using the Mut Express II Mutagenesis Kit (Vazyme, c214, China). *PANK2* frameshift variants, E149X and R490fs494X, were generated by directly cloning their corresponding truncated *PANK2* transcripts into the FUGW-EGFP plasmid. All the mutations were confirmed by Sanger sequencing. Mutation-generated primers for each *PANK2* variant are given in Supplementary Table 1.

### Cell Culture and Transfection

The human embryonic kidney (HEK) 293T human cell line was obtained from the Cell Bank of the Chinese Academy of Sciences (Shanghai, China) and was cultured at 37°C in 5% CO_2_ in Dulbecco’s Modified Eagle’s Medium supplemented with 10% fetal bovine serum (Gibco, cat 10270-106) and 50 U/ml penicillin-streptomycin (Gibco, #15070063). The FUGW-PANK2-EGFP plasmid was transfected into HEK293T cells using the Turbofect Transfection Reagent (Thermo Fisher Scientific, #R0531) according to the manufacturer’s instructions. After 48 h transfection, the cells were subjected to Western blotting or immunofluorescence analysis.

### Western Blotting

To detect soluble expression of wild-type (WT) and mutant PANK2, cell samples were lysed with 1% Triton X-100 lysis buffer [20 mM Tris pH 7.5, 150 mM NaCl, 1% Triton X-100, 2 mM Na_3_VO_4_, 10% β-glycerophosphate, 1 mM ethylene glycol-bis(2-aminoethylether)-N,N,N′,N′-tetraacetic acid (EGTA), 0.5% deoxycholate, and 1 mM phenylmethanesulfonyl fluoride (PMSF)] and centrifuged with 14,000 *g* for 10 min at 4°C. Protein quantification was performed using BCA reagents (Beyotime, China, cat P0012S). A total of 30 μg of protein was loaded onto a 10% polyacrylamide gel and transferred onto a 0.22 μm polyvinylidene difluoride (PVDF) membrane. The membrane was blocked with 5% skimmed milk in tris buffered saline with tween-20 (TBST) buffer (10 mM Tris-HCl pH 8.0, 150 mM NaCl, and 0.05% Tween 20) for 1 h at room temperature. After blocking, the PVDF membrane was incubated overnight with human anti-GFP antibody (#2956, Cell Signaling Technology, 1:1,000) and β-actin (#4970, Cell Signaling Technology, 1:1,000) in an incubation solution at 4°C. After three washes with TBST buffer, the membrane was incubated with horseradish peroxidase (HRP)-conjugated secondary antibody (#7074, Cell Signaling Technology, 1:2,000), diluted in 5% skimmed milk buffer, for 1 h at room temperature. Finally, the membrane was washed three times with TBST buffer and the blots were imaged with a Chemidoc Touch (Bio-Rad) using a chemiluminescent HRP substrate (Bio-Rad).

### Mutant PANK2 Protein Three-Dimensional Modeling Analysis

Pantothenate kinase 2 structural three-dimensional (3D)modeling was performed based on the Protein Data Bank (PDB)^1^ accession (5E26). Analysis of WT and mutant protein modeling was performed using the Iterative Threading Assembly Refinement (I-TASSER) software. The three-dimensional structural images were visualized using PyMOL1.7.

### Mitochondrial Membrane Potential Assay

Mitochondrial membrane potential is an important indicator of normal mitochondrial function and was detected by the MMP indicator, tetramethylrhodamine (TMRM) (#I34361, Invitrogen). Briefly, cells were transfected with WT or mutant *PANK2* plasmids and after 48 h, they were stained with 50 nM TMRM indicator at 37°C under normal culture conditions for 30 min. After the cells were washed with PBS solution, the fluorescence ratio of TMRM/EGFP was measured using the Spectra Max Paradigm Multi-Mode Microplate Reader (Molecular Devices).

### Genotype–Phenotype Analysis

To explore the genotype–phenotype association, publications on *PANK2* mutations and related phenotypes were retrieved from PubMed,^2^ CNKI,^3^ Varcards,^4^ and HGMD^5^ until January 2022. All the *PANK2* variants were annotated based on the transcript NM_153638.4. A LoF mutation is defined as a non-sense, frameshift, canonical splice site, or initiation codon lost mutation. Detailed clinical features of patients with PKAN, including age of onset, GD, dystonia (limbs/oromandibular/generalized), tremor, Parkinsonism, dysarthria, dysphagia, pyramidal signs, MRI, and cognitive decline, were included as described in the literature.

### Statistical Analysis

Statistical analyses were performed using SPSS version 18 (SPSS Incorporation, Chicago, IL, United States). All the quantified data were presented as median (min-max). The *t*-tests, one-way ANOVA tests, and the Kruskal–Wallis tests were used to compare two independent or paired samples, multiple samples, and non-parametric data, respectively. Statistical significance was set at *p* < 0.05.

## Results

### Clinical Features of Seven Patients

A total of seven patients with PKAN were recruited, including three early-onset patients (3/7, 42.9%) and four-late onset patients (4/7, 57.1%). The median age of onset of the patients was 10.0 years old (4.0–22.0). All the patients showed an eye-of-the-tiger sign on T2-weighted MRI. Dysarthria was the most common feature (6/7, 85.7%). Other features present in the patients included gait disturbance (5/7, 71.4%), dystonia (5/7, 71.4%), chorea (4/7, 57.1%), Parkinsonism (2/7, 28.6%), dysphagia (1/7, 14.3%), cognitive decline (1/7, 14.3%), and pyramidal signs (1/7, 14.3%) (Tables 1, 2).

**TABLE 1:** Genetics and clinical features of seven patients with pantothenate kinase 2 (*PANK2*) mutation.

Patient ID	Mutation (origin)	Age at onset (years)/Sex	GD	Dystonia	Tremor	Chorea	Pakinsonism	Dysarthria	Dysphagia	Cognitive Decline	Pyramidal Signs	Eye-of-tiger	Category
1	c.445G > T/p. E149X (P) c.1133A > G/p. D378G (M)	20/Female	–	Focal	–	–	–	+	–	–	–	+	Late-onset
2	c.970G > T/p. D324Y (P) c.970G > T/p. D324Y (M)	10/Female	+	General	–	+	–	–	–	–	–	+	Late-onset
3	c.1103A > G/p. D368G* (P) c.1103A > G/p. D368G* (M)	4/Female	+	Focal	–	+	–	+	–	+	–	+	Early-onset
4	c.1133A > G/p. D378G (P) c.1499A > T/p. N500I (M)	22/Male	+	–	–	–	+	+	–	–	–	+	Late-onset
5^#^	c.1355A > G/p. D452G c.1355A > G/p. D452G	6/Female	+	General	–	+	–	+	–	–	–	+	Early-onset
6	c.1355A > G/p. D452G (P) c.1696C > G/p. L566V* (M)	16/Male	–	–	–	–	+	+	–	–	+	+	Late-onset
7^#^	c.1470delC/p. R490fs494X* c.1470delC/p. R490fs494X*	7/Female	+	General	–	+	–	+	+	–	–	+	Early-onset

P, paternal; GD, Gait Disturbance; M, maternal; +, positive; -, negative; *, novel mutations identified in this study; #, Consanguineous Marriage.

**TABLE 2:** Incidence of clinical features present in patients with pantothenate kinase-associated neurodegeneration (PKAN).

Clinical features	Our cohorts (n = 7)	Pooled (n = 255)
Age of disease onset	10.0 (4.0–22.0)	8.0 (0.3–51.0)
Age at loss of gait	–	11.0 (2.0–60.0)
Early onset	42.9% (3/7)	56.9% (145/255)
Late onset	57.1% (4/7)	43.1% (110/255)
Eye of tiger	100.0% (7/7)	92.7% (217/234)
Dysarthria	85.7% (6/7)	84.0% (157/187)
Gait disturbance	71.4% (5/7)	92.5% (111/120)
Dystonia	71.4% (5/7)	96.7% (235/243)
Chorea	57.1% (4/7)	–
Tremor	–	54.5% (24/44)
Parkinsonism	28.6% (2/7)	51.4% (57/111)
Dysphagia	14.3% (1/7)	54.9% (50/91)
Cognitive decline	14.3% (1/7)	63.9% (46/72)
Pyramidal signs	14.3% (1/7)	61.7% (100/162)
Retinal degeneration	–	47.1 (66/140)
Dystonic opisthotonos	–	79.2% (19/24)

*A total of 255 patients from the publications and our cohorts were pooled analysis. Age of disease onset and at the loss of gait was presented as Median (Min–Max) (years).*

### Identification and Analysis of *PANK2* Mutation

**FIGURE 1:**
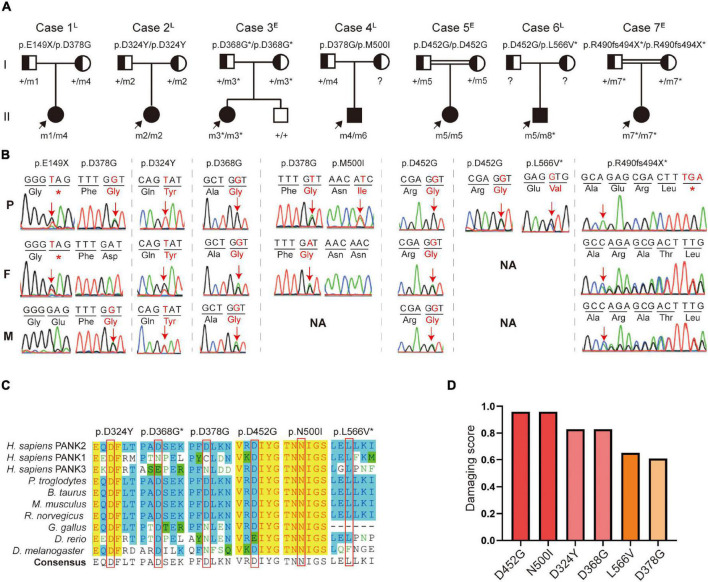
Identification of pantothenate kinase 2 (*PANK2*) mutations in seven unrelated families. **(A)** Pedigrees of the seven patients with *PANK2* mutations. Arrow indicates a proband; asterisk indicates novel mutations found in this study. **(B)** Sanger sequencing chromatogram of the seven probands. Red arrows and alphabets indicate the mutation sites. NA, not available. **(C)** Amino acid sequence alignment shows that D324, D452, L566, and N500 are highly conserved both across species and in pantothenate kinase-associated neurodegeneration (PANK) family proteins; D368 and D378 are highly conserved across species. **(D)** Pathogenicity prediction of six missense mutations using 23 *in silico* predictive algorithms (http://varcards.biols.ac.cn/); the damaging score is shown as the percentage of “damage prediction” of the 23 algorithms. ^E^, early onset; ^L^, late onset.

A total of eight *PANK2* mutations, including six missense and two truncated mutations, were identified in the seven patients. Three novel mutations (c.1103A > G/p.D368G, c.1470delC/p.R490fs494X, and c.1696C > G/p.L566V) were absent in the gnomAD database (https://gnomad.broadinstitute.org). Three homozygous mutations (D368G/D368G, D452G/D452G, and R490fs494X/R490fs494X) were present in the early-onset patients (P3, P5, and P7), including two from consanguineous families. The other four biallelic mutations (D324Y/D324Y, E149X/D378G, D368G/N500I, and D452G/L566V) were found in late-onset patients (P1, P4, and P6) (Figures 1A,B).

All the missense mutations were located on a site that is highly conserved among PANK family proteins and across different species (Figure 1C). These were predicted and evaluated as damage or pathogenic mutations by 23 *in silico* predictive algorithms^6^ and by the American College of Medical Genetics and Genomics (ACMG) guidelines (Richards et al., 2015), respectively (Figure 1D and Supplementary Tables 2, 3). The c.1355A > G (p.D452G) mutation, present in 2/7 unrelated patients (28.6%), was identified as one of the hot spot mutations in the Chinese population (Supplementary Figure 1).

**FIGURE 2:**
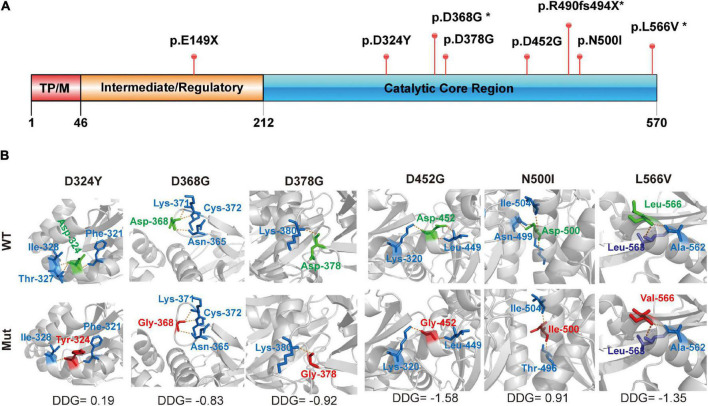
Structural modeling of PANK2 mutants. **(A)** The PANK2 protein mainly contains three domains, namely transit peptide/mitochondrial domain (TPM) in the N-terminal (1–46), intermediate/regulatory domain in the central region (47–243), and catalytic enzyme domain in the C-terminal (244–570). E149X occurring in the intermediate/regulatory domain result in a truncated PANK2, the other PANK2 mutations located in the catalytic enzyme domain. **(B)** Schematic illustration of mutants and their interactions with surrounding amino acids. Red, the mutant amino acids; Green, their surrounding amino acids; Yellow, hydrogen bond. Stability of PANK2 mutant proteins was calculated by I-Mutant suite and shown as ΔΔG (ΔG_Mutant_ -ΔG _WT_) in kcal/mole (ΔΔG < 0 means decreased stability; ΔΔG > 0 means increased stability; an absolute value of ΔΔG above 0.5 means a large decrease/increase of stability).

Two truncated mutations were located in the I/R and CCR domains, which were predicted to produce full or partial CCR loss in PANK2 (Figure 2A). The six missense mutations were located in the CCR of PANK2 and three were predicted to decrease the native hydrogen bonds with surrounding amino acids by using 3-D structural modeling, resulting in varied alterations in the stability of PANK2 (Figure 2B). These results suggest that these mutations would cause PANK2 dysfunction.

### Biallelic Mutation of PANK2 Changed Protein Expression and Disturbed the Mitochondrial Function

**FIGURE 3:**
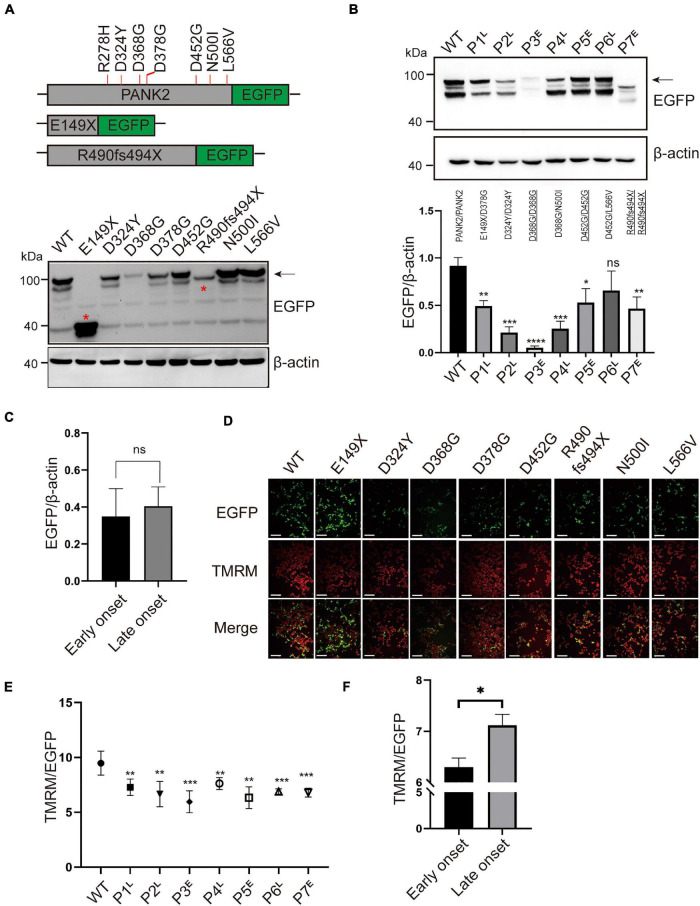
*PANK2* mutants disturbed the protein expression and mitochondrial membrane potential (MMP). **(A)** Recombinant plasmids of PANK2 mutants fused with enhanced green fluorescent protein (EGFP) tag in the C terminus and expressed in HEK 293T cells. Red asterisks indicate the truncated PANK2 protein caused by E149X and R490fs494X mutants. The exogenous protein level of wild type (WT) and mutant PANK2 was determined by immunoblotting using anti-EGFP antibody (β-actin as the internal control). **(B)** Expression of biallelic mutations of *PANK2* in patients was assayed by immunoblotting and was found to be significantly decreased over those of WT. **(C)** Mutated PANK2 expression in patients with early-onset (p3, p5, and p7) and late-onset (p1, p2, p4, and p6) PKAN. **(D)** MMP of HEK 293T cells transfected with WT or PANK2 mutants was detected by TMRM staining, scale bar, 75 μm. **(E)** Compared to WT, biallelic mutant PANK2 decreased MMP significantly. **(F)** Mutated PANK2 from patients with early-onset and late-onset PKAN disturbed the MMP of HEK 293T cells (*p < 0.05; ^**^p < 0.01; ^***^p < 0.001; ^****^p < 0.0001; ns, no significance; t-test, at least three independent experiments).^E^, early onset; ^L^, late onset.

To investigate the altered expression of the biallelic mutations, we first detected the protein expression of WT and mutant PANK2 using immunoblotting. The results showed that the non-sense (E149X) and frameshift (R490fs494X) mutants produced a shorter protein than the WT and missense mutants (Figure 3A). Next, we transfected plasmids containing two mutations at 1:1 into HEK 293T cells, mimicking the biallelic mutations that occurred in the patients. Compared to the WT, six pairs of mutations significantly reduced the PANK2 expression [P1/WT, *t*(4) = 6.976, *p* < 0.01; P2/WT, *t*(4) = 11.47, *p* < 0.001; P3/WT, *t*(4) = 16.68, *p* < 0.0001; P4/WT, *t*(4) = 9.636, *p* < 0.001; P5/WT, *t*(4) = 3.898, *p* < 0.05; and P7/WT, *t*(4) = 5.147, *p* < 0.01] (Figure 3B). There was no difference in the average PANK2 protein expression between the plasmids relating to the early-onset and late-onset patients [*t* (5) = 0.3119, *p* = 0.7677, *t*-test] (Figure 3C).

Pantothenate kinase 2 catalyzes CoA biosynthesis in mitochondria to maintain normal MMP, which is essential for mitochondrial function (Leonardi et al., 2007). We detected cell MMP following WT and mutant plasmid transfection in HEK 293T cells. Compared to the WT, all the pairs of mutations decreased the MMP significantly, causing different degrees of mitochondrial dysfunction [P1/WT, *t*(8) = 3.698, *p* < 0.01; P2/WT, *t*(8) = 3.962, *p* < 0.01; P3/WT, *t*(8) = 5.329, *p* < 0.001; P4/WT, *t*(8) = 3.393, *p* < 0.01; P5/WT, *t*(8) = 4.750, *p* < 0.01; P6/WT, *t*(8) = 5.137, *p* < 0.001; and P7/WT, *t*(8) = 5.761, *p* < 0.001] (Figures 3D, E). Moreover, the average level of MMP damage due to pair mutations derived from the early-onset patients with PKAN was larger than that of the late-onset patients [*t*(6) = 3.906, *p* < 0.05, *t*-test] (Figure 3F). The MMP of the novel homozygous mutation, D368G, was the lowest (Figure 3E). The patient with homozygous mutations manifested the earliest onset age among the seven patients (Table 1). These results indicate that the severity of mitochondrial dysfunction caused by *PANK2* mutations may correlate with the severity of the phenotype of patients.

### Genotype–Phenotype Relationship of PANK2 in PKAN

To further explore the potential relationship between the genotype and phenotype, we analyzed the association between *PANK2* mutation types (homozygous vs. compound heterozygous mutations, biallelic missense vs. biallelic LoF), the PANK2 domain where the mutation occurs (TPM, I/R, CCR), and the phenotype (age of onset, age of lost gait) of patients with PKAN. A total of 255 patients, including 145 (145/255, 56.9%) early onset and 110 (110/255, 43.1%) late onset, were enrolled (seven from this study and 248 from other publications; Supplementary Table 4). The dominant clinical manifestations included eye-of-the-tiger sign in the brain MRI, dysarthria, GD, and dystonia. The median age at onset was 8.0 (0.3–51.0) and the median age at loss of gait was 11.0 (2.0–60.0) (Table 2).

A total of 158 mutations were included in this study, which were grouped into missense (98/158, 62.0%) and LoF mutations (including indels, 36/158, 22.8%; splice site mutations, 11/158, 7.0%; non-senses, 13/158, 8.2%). The ratio of biallelic LoF mutations in early-onset patients was larger than that in late-onset patients (44/50 vs. 6/50, *p* = 4.820 × 10^–6^), which is consistent with previous reports (Chang et al., 2020). There was no difference in the ratio of biallelic missenses or missense/LoF mutations between the two groups of patients (Table 3).

**TABLE 3:** The proportion of 158 *PANK2* mutations in 255 patients with PKAN.

Single mutations	Total percentage		
**Type**			
Missenses	62.0% (98/158)		
Indels	22.8% (36/158)		
Splice site mutations	7.0% (11/158)		
Non-senses	8.2% (13/158)		
**Occurred locations**			
TPM	4.4% (7/158)		
I/R	17.7% (28/158)		
CCR	77.9% (123/158)		

**Bi-allelic mutations**	**Early onset/total patients**	**Late onset/total patients**	**p values**

**Destructive mutations (n = 50)**			
HDMs	42/48	6/48	**8.306 × 10^–6^**
CHDMs	2/2	0/2	/
HDMs + CHDMs	44/50	6/50	**4.820 × 10^–6^**
**Missenses (n = 147)**			
HMMs	42/85	43/85	1.000
CHMMs	29/62	33/62	0.756
HMMs + CHMMs	71/147	76/147	0.762
TPM/TPM	4/4	0/4	/
I(R)/I(R)	4/5	1/5	/
CCR/CCR	63/136	73/136	0.529
I(R)/CCR	0/2	2/2	/
**Destructive/Missense mutations (n = 58)**			
CHMDMs	30/58	28/58	0.873
**Total**	145/255	110/255	0.078

*TPM, transit peptide/mitochondrial domain; I(R), intermediate/regulatory domain; CCR, catalytic core region; HDMs, homozygous destructive mutations; CHDMs, compound heterozygous destructive mutations; HMMs, homozygous missense mutations; CHMMs, compound heterozygous missense mutations; CHMDMs, compound heterozygous missense destructive mutations. Bold integer shows the patient numbers, bold p value indicates a significant difference.*

**FIGURE 4:**
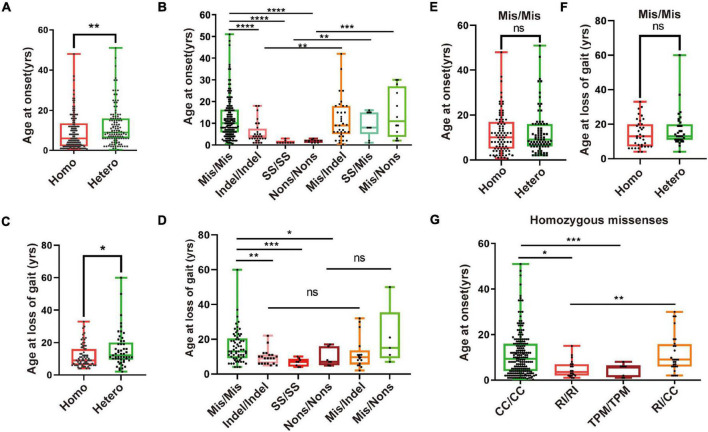
Severity of PKAN may relate to mitochondrial function. **(A)** A total of 255 patients were grouped as those with compound heterozygous (Comhet) (122/255, 47.8%) and those with homozygous (Homo) (133/255, 52.2%) mutations of *PANK2*. Patients with homozygous mutations have an earlier age of onset than those with heterozygous mutations [6.0 (0.7–48.0) vs. 9.3 (0.3–51.0)]. **(B)** Two hundred and fifty patients with biallelic *PANK2* mutations were divided into 7 groups, namely missense/missense (Mis/Mis, *n* = 147), indel/indel (*n* = 28), splice-site mutation/splice-site mutation (SS/SS, *n* = 9), non-sense/non-sense (Nons/Nons, *n* = 11), missense/indel (Mis/indel, n = 37), splice-site mutation/missense (SS/Mis, *n* = 9), and missense/non-sense (Mis/Nons, *n* = 12). Differences across the groups were analyzed. **(C)** Difference of age at loss of gait between patient with homozygous *PANK2* mutation (*n* = 71) and that with heterozygous *PANK2* mutations (*n* = 53) [9.0 (4.0–33.0) vs. 13.0 (2.0–50.0)]. **(D)** A total of 122 patients with the information of age at loss of gait were grouped into Mis/Mis (*n* = 65), indel/indel (*n* = 20), SS/SS (*n* = 8), Nons/Nons (*n* = 7), Mis/indel (*n* = 17), and Mis/Nons (*n* = 5) to value the severity of PKAN. Age at onset **(E)** and loss of gait **(F)** of patients with heterozygous and homozygous Mis/Mis were analyzed. **(G)** One hundred and forty-six patients with missense mutations were further divided into four groups, namely TPM/TPM (n = 8), R (I)/R (I) (n = 20), CC/CC (*n* = 109), and R (I)/CC (*n* = 9). Difference across the groups was analyzed using the Mann–Whitney *U* test (*p < 0.05, **p < 0.01, ***p < 0.001, ****p < 0.0001; ns, no significance, the Mann–Whitney *U* test).

Next, we found that the age of onset and loss of gait for patients with homozygous mutations was younger than that of patients with compound heterozygous mutations (Figures 4A,C). Furthermore, we found that patients with homozygous LoF mutations have an earlier onset age and loss of gait than those with homozygous or heterozygous missense/LoF mutations (Figures 4B,D). Although there was no difference in the age of onset and at loss gait between the patients with the homozygous and those with compound heterozygous missense mutations (Figures 4E,F), the patients with the missense mutation that occurred in the TPM domain had an earlier age of onset than those with mutations in the other two domains (Figure 4G). Taken together, these results indicate that the degree of mitochondrial damage caused by biallelic mutations in *PANK2* could be related to the severity of PKAN.

## Discussion

Pantothenate kinase-associated neurodegeneration is an autosomal recessive disorder caused by biallelic variation in *PANK2*, which encodes a mitochondrial protein implicated in the biosynthesis of CoA (Hartig et al., 2006; Efthymiou et al., 2020). In this study, we identified eight *PANK2* mutations in seven unrelated families, including three novel mutations (c.1103A > G/p.D368G, c.1470delC/p.R490fs494X, and c.1696C > G/p.L566V). Most pair mutations of *PANK2* (6/7, 85.7%) decreased PANK2 protein expression and impaired MMP in HEK 293T cells. Further systematic analysis revealed that patients with an LoF mutation that completely disrupted the mitochondrial function of PANK2 or a missense mutation occurring in its mitochondrial transit peptide domain had a tendency toward a more severe phenotype. These results indicate that *PANK2* mutations contribute to the patient phenotype by disturbing mitochondrial function.

Human PANK2 protein, a homodimer, catalyzes the rate-limiting first step of CoA biosynthesis in a feedback-regulation manner (Kotzbauer et al., 2005). PANK2 is highly expressed in the brain and is localized in the mitochondrial inner membrane (Zhou et al., 2001; Kotzbauer et al., 2005). Silencing of PANK2 expression causes cell growth reduction, cell-specific ferroprotein upregulation, and iron deregulation in human cell lines, characteristics associated with the pathological features of neurodegeneration associated with brain iron accumulation in the basal ganglia (Kruer et al., 2011). LoF *PANK2* mutations perturb mitochondrial function and iron homeostasis in mouse and human cells (Santambrogio et al., 2015; Orellana et al., 2016; Jeong et al., 2019). Similarly, in our cohort, biallelic mutations in *PANK2* decreased PANK2 protein levels and damaged mitochondrial function. Furthermore, the disruption of mitochondrial function, but not PANK2 expression, was significantly high for mutations found in early-onset patients (Figures 3C,F). Previous studies supported that variable functional alteration resulting from different mutations in a same gene led to varied severity phenotype (Cai et al., 2019; Shi et al., 2019; Wang et al., 2020, 2021b). In our pooled analysis of 255 patients, the patients with LoF mutations exhibited a more severe phenotype. It is worth noting that patients with a missense mutation that occurs in the PANK2 TPM domain also displayed a more severe phenotype than those with mutations in the other two domains.

The TPM domain is important for PANK2 function as an acylcarnitine sensor that upregulates CoA biosynthesis (Brunetti et al., 2012) and we propose that missense mutations in the TPM domain lead to the loss of PANK2 function by disturbing its translocation into mitochondria. These results indicated that biallelic mutations leading to impaired mitochondrial function or disturbing PANK2 trafficking into the mitochondria may result in an earlier onset of PKAN.

Coenzyme A is a crucial molecule participating in more than 100 metabolic processes (Siudeja et al., 2011; Wang et al., 2019). PANK2 catalyzes the synthesis of CoA from pantothenate (vitamin B_5_) in the mitochondrial intermembrane space and acts as a sensor for mitochondrial CoA requirement and a regulator of CoA biosynthesis (Brunetti et al., 2012; Zizioli et al., 2016). Supplementation of CoA in human induced pluripotent stem cells (iPSC)-derived neurons with PANK2 deficiency was sufficient to rescue the majority of mitochondrial functionally defective phenotypes of PKAN (Orellana et al., 2016). In this study, we found that the patients with the mutation located in the mitochondrial harboring domain (TPM) of PANK2 displayed an earlier age of onset, indicating that they could benefit from earlier supplementation of CoA for improved PKAN treatment.

In addition, the frequency of mutation sites of PANK2 between Chinese and other populations was different. The top three of the most frequent mutations in the Chinese population were p.D378G (13/120), p.D452G (7/120), and p.I501T (7/120). While the top three of the most frequent mutations in other populations were p.N404I (20/378), p.G219S/V (15/204), and p.T528M (12/204). Some mutations only occurred in the Chinese population, such as p.D452G and p.D324Y (Supplementary Figure 1). These differences may be attributed to the founder effect (Rump et al., 2005; Nakatsuka et al., 2017).

In conclusion, this study identified three novel *PANK2* mutations in seven PKAN families and found a correlation between genotype and mitochondrial function impairment and phenotype. Our results may provide a prediction of the severity of this disease and may provide insights for recognizing the disease mechanism of PKAN.

## Data Availability Statement

The datasets presented in this study can be found in online repositories. The names of the repository/repositories and accession number(s) can be found in the article/Supplementary Material.

## Ethics Statement

The study was approved by the Medical Ethics Committee of Second Affiliated Hospital of Guangzhou Medical University. Written informed consent to participate in this study was provided by the participants’ legal guardian/next of kin.

## Author Contributions

Y-WS, X-WS, and W-BL defined the research theme and wrote the manuscript. W-BL, N-XS, H-CX, Z-YL, LC, C-XF, and QC designed methods and experiments and carried out most of the experiments. X-WS, N-XS, CZ, and LC collected the clinic data. W-BL and N-XS analyzed the data and interpreted the results. All authors listed have made a substantial, direct, and intellectual contribution to the work, and approved it for publication.

## Conflict of Interest

The authors declare that the research was conducted in the absence of any commercial or financial relationships that could be construed as a potential conflict of interest.

## Publisher’s Note

All claims expressed in this article are solely those of the authors and do not necessarily represent those of their affiliated organizations, or those of the publisher, the editors and the reviewers. Any product that may be evaluated in this article, or claim that may be made by its manufacturer, is not guaranteed or endorsed by the publisher.

## Footnotes

1
http://www.rcsb.org

2
https://pubmed.ncbi.nlm.nih.gov/

3
https://www.cnki.net/

4
http://varcards.biols.ac.cn/

5
http://www.hgmd.cf.ac.uk/ac/index.php

6
http://varcards.biols.ac.cn/

## Supplementary Material

Supplementary Figure 1 Reoccurring number and distribution of 158 pantothenate kinase 2 *(PANK2)* mutations in patients with pantothenate kinase-associated neurodegeneration (PKAN) in China and other countries. Hotspot mutations were p.N404I and p.D378G in all patients with PKAN and Chinese populations, respectively.

Supplementary Table 1 Primers used for plasmid construction and mutagenesis.

Supplementary Table 2 Pathogenic prediction of 6 missense mutations of PANK2 by 23 *in silico* predictive algorithms.

Supplementary Table 3 Pathogenic prediction of PANK2 mutations with the American College of Medical Genetics and Genomics (ACMG).

Supplementary Table 4 General information regarding 255 patients with PKAN.
