# Supplementary material for: Novel PANK2 Mutations in Patients With Pantothenate Kinase-Associated Neurodegeneration and the Genotype–Phenotype Correlation
Source: Front Aging Neurosci. 2022 Apr 6;14:848919. doi: 10.3389/fnagi.2022.848919 (PMC9019683; doi:10.3389/fnagi.2022.848919)
Supplement: Supplementary file 1 [file Data_Sheet_1.PDF]

References of Supplementary Table 4

1. Gonzalez Huerta, L. M., Gomez Gonzalez, S. & Toral Lopez, J. Psychiatric symptoms in an adolescent reveal a novel compound heterozygous mutation of the PANK2 gene in the atypical PKAN syndrome. Psychiatr Genet 31, 95-99, doi:10.1097/YPG.0000000000000278 (2021).
2. Yang, F., Wang, J., Yang, Z., Ren, Z. & Zeng, F. PANK2 p. A170fsa novel pathogenetic mutation, compound with PANK2 p.R440P, causing pantothenate kinase Associated neurodegeneration in a Chinese family. Int J Neurosci, 1-7, doi:10.1080/00207454.2020.1828883 (2020).
3. Chang, X., Zhang, J., Jiang, Y., Wang, J. & Wu, Y. Natural history, and genotype-phenotype correlation of pantothenate kinase-associated neurodegeneration. CNS Neurosci Ther 26, 754-761, doi:10.1111/cns.13294 (2020).
4. Zhang, Y., Zhou, D. & Yang, T. Novel PANK2 mutation in a Chinese boy with PANK2-associated neurodegeneration: A case report and review of Chinese cases. Medicine (Baltimore) 98, e14122, doi:10.1097/MD.0000000000014122 (2019).
5. Sakpichaisakul, K. et al. Novel PANK2 mutation discovered among South East Asian children living in Thailand affected with pantothenate kinase associated neurodegeneration. J Clin Neurosci 66, 187-190, doi: 10.1016/j.jocn.2019.04.017 (2019).
6. Habibi, A. H. et al. A novel homozygous variation in the PANK2 gene in two Persian siblings with atypical pantothenate kinase associated neurodegeneration. Neurol Int 11, 7959, doi:10.4081/ni.2019.7959 (2019).
7. Shi, X. et al. Basal ganglia calcification and novel compound heterozygous mutations in the PANK2 gene in a Chinese boy with classic Pantothenate kinase-associated neurodegeneration: A case report. Medicine (Baltimore) 97, e0316, doi:10.1097/MD.0000000000010316 (2018).
8. Cheng, Y. et al. Novel compound heterozygous PANK2 gene mutations in a Chinese patient with atypical pantothenate kinase-associated neurodegeneration. Int J Neurosci 128, 1109-1113, doi:10.1080/00207454.2018.1483364 (2018).
9. Rohani, M. et al. Tremor-Dominant Pantothenate Kinase-associated Neurodegeneration. Mov Disord Clin Pract 4, 772-774, doi:10.1002/mdc3.12512 (2017).
10. Paraskevas, G. P. et al. Novel PANK2 mutation in the first Greek compound heterozygote patient with pantothenate-kinase-associated neurodegeneration. SAGE Open Med Case Rep 5, 2050313X17720101, doi:10.1177/2050313X17720101 (2017).
11. Israni, A. V. & Mandal, A. Atypical pantothenate kinase-associated neurodegeneration with novel genetic mutation. Neurol India 65, 914-915, doi:10.4103/neuroindia.NI_1260_16 (2017).
12. Dastsooz, H., Nemati, H., Fard, M. A. F., Fardaei, M. & Faghihi, M. A. Novel mutations in PANK2 and PLA2G6 genes in patients with neurodegenerative disorders: two case reports. BMC Med Genet 18, 87, doi:10.1186/s12881-017-0439-y (2017).
13. Angural, A. et al. A variation in PANK2 gene is causing Pantothenate kinase-associated Neurodegeneration in a family from Jammu and Kashmir - India. Sci Rep 7, 4834, doi:10.1038/s41598-017-05388-9 (2017).
14. Akcakaya, N. H. et al. Clinical and genetic features of PKAN patients in a tertiary centre in Turkey. Clin Neurol Neurosurg 154, 34-42, doi: 10.1016/j.clineuro.2017.01.011 (2017).
15. Yapici, Z., Akcakaya, N. H., Tekturk, P., Iseri, S. A. & Ozbek, U. A novel gene mutation in PANK2 in a patient with severe jaw-opening dystonia. Brain Dev 38, 755-758, doi: 10.1016/j.braindev.2016.02.010 (2016).

16. Li, Y. F., Li, H. F., Zhang, Y. B. & Wu, J. M. Novel homozygous PANK2 mutation identified in a consanguineous Chinese pedigree with pantothenate kinase-associated neurodegeneration. Biomed Rep 5, 217-220, doi:10.3892/br.2016.715 (2016).
17. Lee, J. H. et al. Clinical Heterogeneity of Atypical Pantothenate Kinase-Associated Neurodegeneration in Koreans. J Mov Disord 9, 20-27, doi:10.14802/jmd.15058 (2016).
18. Han, J., Kim do, W., Lee, C. H. & Han, S. H. Optic Atrophy in a Patient With Atypical Pantothenate Kinase-Associated Neurodegeneration. J Neuroophthalmol 36, 182-186, doi:10.1097/WNO.0000000000000335 (2016).
19. Ghafouri-Fard, S. et al. A Novel Nonsense Mutation in PANK2 Gene in Two Patients with Pantothenate Kinase-Associated Neurodegeneration. Int J Mol Cell Med 5, 255-259 (2016).
20. Tanrikulu, B. et al. Deep brain stimulation as treatment for dystonic storm in pantothenate kinase-associated neurodegeneration syndrome: case report of a patient with homozygous C.628 2 T > G mutation of the PANK2 gene. Acta Neurochir (Wien) 157, 1513-1516; discussion 1516-1517, doi:10.1007/s00701-015-2514-5 (2015).
21. Morales-Briceno, H. et al. Clinical, imaging, and molecular findings in a sample of Mexican families with pantothenate kinase-associated neurodegeneration. Clin Genet 87, 259-265, doi:10.1111/cge.12400 (2015).
22. Ma, L. Y. et al. Novel gene mutations and clinical features in patients with pantothenate kinase-associated neurodegeneration. Clin Genet 87, 93-95, doi:10.1111/cge.12341 (2015).
23. Tanteles, G. A., Spanou-Aristidou, E., Antoniou, C., Christophidou-Anastasiadou, V. & Kleopa, K. A. Novel homozygous PANK2 mutation causing atypical pantothenate kinase-associated neurodegeneration (PKAN) in a Cypriot family. J Neurol Sci 340, 233-236, doi: 10.1016/j.jns.2014.03.001 (2014).
24. Shan, J. et al. Novel PANK2 gene mutations in two Chinese siblings with atypical pantothenate kinase-associated neurodegeneration. Neurol Sci 34, 561-563, doi:10.1007/s10072-012-1177-8 (2013).
25. Perez-Gonzalez, E. A., Chacon-Camacho, O. F., Arteaga-Vazquez, J., Zenteno, J. C. & Mutchinick, O. M. A novel gene mutation in PANK2 in a patient with an atypical form of pantothenate kinase-associated neurodegeneration. Eur J Med Genet 56, 606-608, doi: 10.1016/j.ejmg.2013.08.007 (2013).
26. Pan, L. S., Yu, L. H., Yin, Y. Y. & Xu, Y. M. A novel PANK2 mutation in a 12-year-old Chinese boy with pantothenate kinase-associated neurodegeneration. Neurol India 61, 175-176, doi:10.4103/0028-3886.111134 (2013).
27. Lee, C. H. et al. Phenotypes and genotypes of patients with pantothenate kinase-associated neurodegeneration in Asian and Caucasian populations: 2 cases and literature review. ScientificWorldJournal 2013, 860539, doi:10.1155/2013/860539 (2013).
28. Hamamoto Filho, P. T. et al. Isolated cortical vein thrombosis in a patient with sickle cell disease: treatment with decompressive craniotomy and anticoagulation and literature review. Neurol India 61, 173-175, doi:10.4103/0028-3886.111132 (2013).
29. Diaz, N. Late onset atypical pantothenate-kinase-associated neurodegeneration. Case Rep Neurol Med 2013, 860201, doi:10.1155/2013/860201 (2013).
30. Aryani, O., Houshmand, M. & Fatehi, F. A novel PANK2 gene mutation in a Persian boy: The first report from Iran. Clinical Neurology and Neurosurgery 115, 1170-1172, doi: 10.1016/j.clineuro.2012.10.004 (2013).

31. Lim, B. C. et al. Pantothenate kinase-associated neurodegeneration in Korea: recurrent R440P mutation in PANK2 and outcome of deep brain stimulation. Eur J Neurol 19, 556-561, doi:10.1111/j.1468-1331.2011. 03589.x (2012).
32. Mak, C. M. et al. Young-onset parkinsonism in a Hong Kong Chinese man with adult-onset Hallervorden-Spatz syndrome. Int J Neurosci 121, 224-227, doi:10.3109/00207454.2010.542843 (2011).
33. Kruer, M. C. et al. Novel histopathologic findings in molecularly-confirmed pantothenate kinase-associated neurodegeneration. Brain 134, 947-958, doi:10.1093/brain/awr042 (2011).
34. Ferini-Strambi, L. et al. Malabsorption is uncommon in restless legs syndrome. Mov Disord 26, 1767-1768, doi:10.1002/mds.23615 (2011).
35. Chiapparini, L. et al. The "eye-of-the-tiger" sign may be absent in the early stages of classic pantothenate kinase associated neurodegeneration. Neuropediatrics 42, 159-162, doi:10.1055/s-0031-1285925 (2011).
36. Camargos, S. T. et al. Low prevalence of PANK2 mutations in Brazilian patients with early onset generalised dystonia and basal ganglia abnormalities on MRI. J Neurol Neurosurg Psychiatry 82, 1059-1060, doi:10.1136/jnnp.2009.200808 (2011).
37. Mehta, S. H. et al. Predominant ataxia, low ceruloplasmin, and absent K-F rings: hypoceruloplasminemia or Wilson's disease. Mov Disord 25, 2260-2261, doi:10.1002/mds.23000 (2010).
38. Doi, H. et al. Siblings with the adult-onset slowly progressive type of pantothenate kinase-associated neurodegeneration and a novel mutation, Ile346Ser, in PANK2: clinical features and (99m) Tc-ECD brain perfusion SPECT findings. J Neurol Sci 290, 172-176, doi: 10.1016/j.jns.2009.11.008 (2010).
39. Aggarwal, A. et al. Indian-subcontinent NBIA: unusual phenotypes, novel PANK2 mutations, and undetermined genetic forms. Mov Disord 25, 1424-1431, doi:10.1002/mds.23095 (2010).
40. Seo, J. H., Song, S. K. & Lee, P. H. A Novel PANK2 Mutation in a Patient with Atypical Pantothenate-Kinase-Associated Neurodegeneration Presenting with Adult-Onset Parkinsonism. J Clin Neurol 5, 192-194, doi:10.3988/jcn.2009.5.4.192 (2009).
41. Kim, S. H. et al. Novel compound heterozygous mutations in the pantothenate kinase 2 gene in a korean patient with atypical pantothenate kinase associated neurodegeneration. J Mov Disord 2, 45-47, doi:10.14802/jmd.09012 (2009).
42. Gardner, R. C., Alcalay, R. N. & Schmahmann, J. D. Transient exacerbation of ataxia with smoking: a prevalence survey. Mov Disord 24, 937-938, doi:10.1002/mds.22470 (2009).
43. Bozi, M., Matarin, M., Theocharis, I., Potagas, C. & Stefanis, L. A patient with pantothenate kinase-associated neurodegeneration and supranuclear gaze palsy. Clin Neurol Neurosurg 111, 688-690, doi: 10.1016/j.clineuro.2009.04.007 (2009).
44. Lyoo, C. H. et al. Anticholinergic-responsive gait freezing in a patient with pantothenate kinase-associated neurodegeneration. Mov Disord 23, 283-284, doi:10.1002/mds.21799 (2008).
45. Chung, S. J., Lee, J. H., Lee, M. C., Yoo, H. W. & Kim, G. H. Focal hand dystonia in a patient with PANK2 mutation. Mov Disord 23, 466-468, doi:10.1002/mds.21880 (2008).
46. Chan, K. Y., Lam, C. W., Lee, L. P., Tong, S. F. & Yuen, Y. P. Pantothenate kinase-associated neurodegeneration in two Chinese children: identification of a novel PANK2 gene mutation. Hong Kong Med J 14, 70-73 (2008).
47. Saleheen, D., Ali, T., Aly, Z., Khealani, B. & Frossard, P. M. Novel mutation in the PANK2

gene leads to pantothenate kinase-associated neurodegeneration in a Pakistani family. Pediatr Neurol 37, 296-298, doi: 10.1016/j.pediatrneurol.2007.05.015 (2007).
48. Zolkipli, Z., Dahmoush, H., Saunders, D. E., Chong, W. K. & Surtees, R. Pantothenate kinase 2 mutation with classic pantothenate-kinase-associated neurodegeneration without 'eye-of-the-tiger' sign on MRI in a pair of siblings. Pediatr Radiol 36, 884-886, doi:10.1007/s00247-006-0205-3 (2006).
49. Surtees, Z. Z. H. D. D. E. S. W. K. K. C. R. Pantothenate kinase 2 mutation with classic pantothenate-kinase-associated neurodegeneration without ‘eye-of-the-tiger’ sign on MRI in a pair of siblings. Pediatr Radiol (2006).
50. Matarin, M. M., Singleton, A. B. & Houlden, H. PANK2 gene analysis confirms genetic heterogeneity in neurodegeneration with brain iron accumulation (NBIA) but mutations are rare in other types of adult neurodegenerative disease. Neurosci Lett 407, 162-165, doi: 10.1016/j.neulet.2006.08.030 (2006).
51. Liang, T. W. et al. Partial deficit of pantothenate kinase 2 catalytic activity in a case of tremor-predominant neurodegeneration with brain iron accumulation. Mov Disord 21, 718-722, doi:10.1002/mds.20797 (2006).
52. Hartig, M. B. et al. Genotypic and phenotypic spectrum of PANK2 mutations in patients with neurodegeneration with brain iron accumulation. Ann Neurol 59, 248-256, doi:10.1002/ana.20771 (2006).
53. Zhang, Y. H. et al. Novel compound heterozygous mutations in the PANK2 gene in a Chinese patient with atypical pantothenate kinase-associated neurodegeneration. Mov Disord 20, 819-821, doi:10.1002/mds.20408 (2005).
54. Rump, P. et al. A novel 3-bp deletion in the PANK2 gene of Dutch patients with pantothenate kinase-associated neurodegeneration: evidence for a founder effect. Neurogenetics 6, 201-207, doi:10.1007/s10048-005-0018-9 (2005).
55. Pellecchia, M. T. et al. The diverse phenotype and genotype of pantothenate kinase-associated neurodegeneration. Neurology 64, 1810-1812, doi: 10.1212/01.WNL.0000161843. 52641.EC (2005).
56. Hajek, M. et al. MR relaxometry and 1H MR spectroscopy for the determination of iron and metabolite concentrations in PKAN patients. Eur Radiol 15, 1060-1068, doi:10.1007/s00330-004-2553-4 (2005).
57. Yamashita, S. et al. Pantothenate kinase-associated neurodegeneration initially presenting as postural tremor alone in a Japanese family with homozygous N245S substitutions in the pantothenate kinase gene. J Neurol Sci 225, 129-133, doi: 10.1016/j.jns.2004.07.012 (2004).
58. Thomas, M., Hayflick, S. J. & Jankovic, J. Clinical heterogeneity of neurodegeneration with brain iron accumulation (Hallervorden-Spatz syndrome) and pantothenate kinase-associated neurodegeneration. Mov Disord 19, 36-42, doi:10.1002/mds.10650 (2004).
59. Vasconcelos, O. M. et al. Adult Hallervorden-Spatz syndrome simulating amyotrophic lateral sclerosis. Muscle Nerve 28, 118-122, doi:10.1002/mus.10389 (2003).
60. Houlden, H. et al. Compound heterozygous PANK2 mutations confirm HARP and Hallervorden-Spatz syndromes are allelic. Neurology 61, 1423-1426, doi: 10.1212/01.wnl.0000094120.09977.92 (2003).
61. 武晔, 周. 贺. 寇. 封. 邓. 周. 王. 姜. 20 例经典泛酸激酶相关神经变性的临床表型和基因型特点. 中华儿科杂志 55, 678-682 (2017).

62. 廖卫平, 宋. 王. 石. 邓. 陈. 林. 易. Hallervorden--Spatz 综合征患者一例的临床 特征及泛酸激酶 2 基因突变检测. 中华医学杂志 89, 3320-3323 (2009).
63. 朱军. 一个泛酸激酶相关的神经变性病家系的临床特征与致病基因分析. 山东大学硕士学位论文 (2011).
64. 晶, 贺. 15 例 PANK2 基因相关疾病的基因检测与临床分析. 山西医科大学硕士学位论文 (2013).
65. 江泓, 张. 唐. 窦. 陈. 陈. 郭. 龙. 夏. 潘. 许. 汤. 严. 沈. Hallervorden-Spatz 综合征的临床、磁共振成像特征及泛酸激酶 2 基因的突变检测. 中华神经科杂志 38, 34-37 (2005).
