# Supplementary figures and images for: Novel PANK2 Mutations in Patients With Pantothenate Kinase-Associated Neurodegeneration and the Genotype–Phenotype Correlation
Source: Front Aging Neurosci. 2022 Apr 6;14:848919. doi: 10.3389/fnagi.2022.848919 (PMC9019683; doi:10.3389/fnagi.2022.848919)

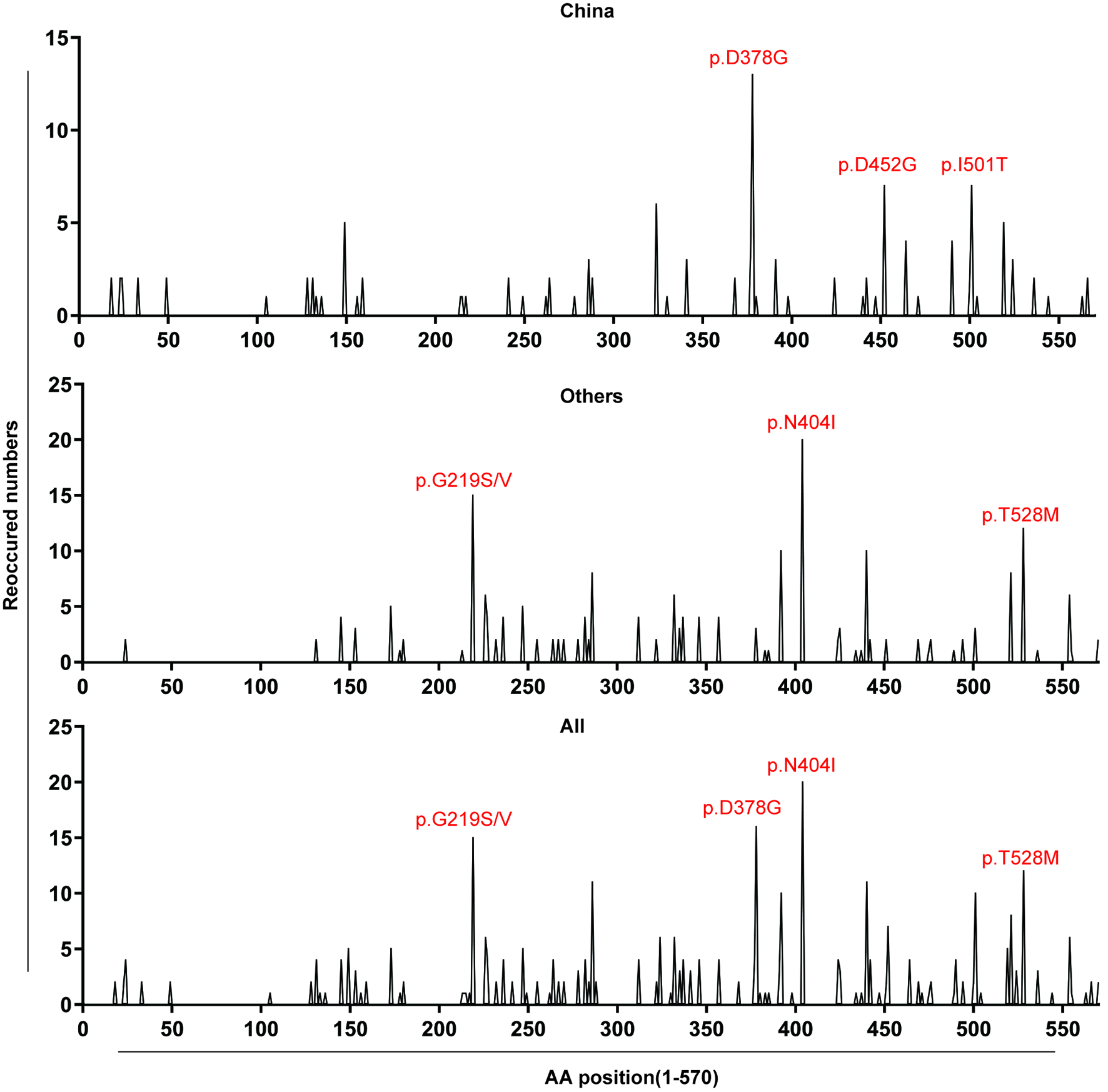

Supplement: Supplementary Figure 1 — Reoccurring number and distribution of 158 pantothenate kinase 2 (PANK2) mutations in patients with pantothenate kinase-associated neurodegeneration (PKAN) in China and other countries. Hotspot mutations were p.N404I and p.D378G in all patients with PKAN and Chinese populations, respectively. [file Image_1.TIF]
