# Supplementary material for: Novel PANK2 Mutations in Patients With Pantothenate Kinase-Associated Neurodegeneration and the Genotype–Phenotype Correlation
Source: Front Aging Neurosci. 2022 Apr 6;14:848919. doi: 10.3389/fnagi.2022.848919 (PMC9019683; doi:10.3389/fnagi.2022.848919)
Supplement: Supplementary Table 1 — Primers used for plasmid construction and mutagenesis. [file Table_1.DOCX]

**Supplementary table 1.** Primers using for plasmids construction and mutagenesis

| Primer IDs | Sequence (5’-3’) |
| --- | --- |
| PANK2-F | AGGATCCCCGGGTACCGGTCGCCACCATGAGGAGGCTC |
| PANK2-R | ATAAGCTTGATATCGAATTCTCACGGGATCTTCAACAGCT |
| PKAN2-445-F | GGCTGCAGGTCGACTCTAGAGCCACCATGAGGAGGCTCGG |
| PANK2-445-R | GCTCGAGATCTGAGTCCGGACCCAGCCGACGAGACGGA |
| PANK2-833-F | GACCTGACTCTGTGTGGACACAAAGGCAATCTGCACTTT |
| PANK2-833-R | AAAGTGCAGATTGCCTTTGTGTCCACACAGAGTCAGGTC |
| PANK2-970-F | GAGCGTACAAATTTGAGCAGTATTTTCTCACAATAGGTG |
| PANK2-970-R | CACCTATTGTGAGAAAATACTGCTCAAATTTGTACGCTC |
| PANK2-1103-F | TACTTTGAAAACCCTGCTGGTTCTGAAAAGTGTCAGAAG |
| PANK2-1103-R | CTTCTGACACTTTTCAGAACCAGCAGGGTTTTCAAAGTA |
| PANK2-1133-F | GTGTCAGAAGTTACCATTTGGTTTGAAAAATCCGTATCC |
| PANK2-1133-R | GGATACGGATTTTTCAAACCAAATGGTAACTTCTGACAC |
| PANK2-1355-F | GTGGATAAACTAGTACGAGGTATTTATGGAGGGGACTATG |
| PANK2-1355-R | CATAGTCCCCTCCATAAATACCTCGTACTAGTTTATCCAC |
| PANK2-1470-F | GTCAGTAAAGAGGACCTGGCAGAGCGACTTTGATCACC |
| PANK2-1470-R | GCTCGAGATCTGAGTCCGGACAGGTCCTCTTTACTGACAGCCTC |
| PANK2-1499-F | CTTTGATCACCATCACCAACATCATTGGCTCAATAGCAAGAATG |
| PANK2-1499-R | CATTCTTGCTATTGAGCCAATGATGTTGGTGATGGTGATCAAAG |
| PANK2-1696-R | ATAAGCTTGATATCGAATTCTCACGGGATCTTCAACACCTCAAGGAG |
