# Supplementary material for: Novel PANK2 Mutations in Patients With Pantothenate Kinase-Associated Neurodegeneration and the Genotype–Phenotype Correlation
Source: Front Aging Neurosci. 2022 Apr 6;14:848919. doi: 10.3389/fnagi.2022.848919 (PMC9019683; doi:10.3389/fnagi.2022.848919)
Supplement: Supplementary Table 3 — Pathogenic prediction of PANK2 mutations with the American College of Medical Genetics and Genomics (ACMG). [file Table_3.DOCX]

**Supplementary Table 3.** Pathogenicity of PANK2 mutations assessed with ACMG guidelines.

| **Nucleotide/Protein change** | **ACMG after our study** |
| --- | --- |
| c.445G>G/p. E149X | Pathogenic (PVS1+PS3) |
| c.833G>A/p. R278H | Pathogenic (PS1+PS3) |
| c.970G>T/p. D324Y | Pathogenic (PS1+PS3) |
| c.1103A>G/p. D368G**^*^** | Pathogenic (PS3+PM2+PM5++PP2+PP3+PP4) |
| c.1133A>G/p. D378G | Pathogenic (PS1+PS3) |
| c.1355A>G/p. D452G | Pathogenic (PS1+PS3) |
| c.1470delC/p. R490fs494X^*^ | Pathogenic (PVS1+PS3) |
| c.1499A>T/p. N500I | Pathogenic (PS1+PS3) |
| c.1696C>G/p. L566V**^*^** | Pathogenic (PS3+PM2+PM5++PP2+PP3+PP4) |

*****, Novel PANK2 mutations found in our study. Pathogenic criterion is weighted as very strong (PVS1), strong (PS1–4); moderate (PM1–6), or supporting (PP1–5) according the guidelines(Richards et al., 2015).
